# Contribution of *C*-glucosidic ellagitannins to *Lythrum salicaria* L. influence on pro-inflammatory functions of human neutrophils

**DOI:** 10.1007/s11418-014-0873-5

**Published:** 2014-10-28

**Authors:** Jakub P. Piwowarski, Anna K. Kiss

**Affiliations:** Department of Pharmacognosy and Molecular Basis of Phytotherapy, Faculty of Pharmacy, Medical University of Warsaw, ul. Banacha 1, 02-097 Warsaw, Poland

**Keywords:** *Lythrum salicaria*, Ellagitannins, Neutrophils, Inflammation

## Abstract

The herb *Lythrum salicaria* L. (Lythraceae) is used in traditional medicine to treat diseases with an inflammatory background, such as haemorrhoidal disease, dysentery, chronic intestinal catarrh, eczema, varicose veins, periodontosis and gingivitis. Because these diseases are closely associated with an excessive inflammatory response of stimulated neutrophils, the influence of aqueous extract and isolated *C*-glucosidic ellagitannins (dimeric salicarinins A, B and C, vescalagin, castalagin) on their pro-inflammatory functions was examined. *Lythrum salicaria* aqueous extract was shown to modulate lipopolysaccharide (LPS)-triggered production of IL-8 (at 20 μg/mL, 16.6 ± 4.2 % inhibition) but had no influence on MMP-9 production. It was active towards cytochalasin A/f-MLP- stimulated elastase release (at 20 μg/mL, 21.5 ± 3.9 % inhibition), myeloperoxidase release (at 1 μg/mL, 26.5 ± 5.4 % inhibition) and f-MLP- and PMA-induced reactive oxygen species production (at 20 μg/mL, 67.0 ± 3.9 and 66.5 ± 1.9 % inhibition, respectively). The extract was also shown to inhibit expression of integrin CD11b on the neutrophil surface without influencing selectin CD62L shedding. Dose-dependent inhibition of hyaluronidase activity was observed with IC_50_ = 10.1 ± 1.2 μg/mL. The main *C*-glucosidic ellagitannins were shown to be responsible for all these activities with more significant participation attributable to dimeric salicarinins A, B, C. This study has demonstrated potent activity of aqueous extract on stimulated neutrophils; this enhanced response is known to cause pathological changes in skin and mucosa tissues. These observations support and explain the traditional use of the herb *Lythrum salicaria* to treat certain diseases with an inflammatory background. *C*-glucosidic ellagitannins, especially dimeric salicarinins, are the factors responsible for these effects.

**Electronic supplementary material:**

The online version of this article (doi:10.1007/s11418-014-0873-5) contains supplementary material, which is available to authorized users.

## Introduction

The herb *Lythrum salicaria* L. has been used in traditional medicine to treat diseases with an inflammatory background, such as haemorrhoidal disease, dysentery, chronic intestinal catarrh, eczema, varicose veins, periodontosis and gingivitis [1–4]. These diseases are closely associated with an excessive inflammatory response and increased extracellular matrix degradation causing impaired tissue integrity. Neutrophils, which enhance the response, are known to cause pathological changes in skin and mucosa tissues and are important contributors to the development of pathological changes during progression of the above diseases. Being responsible for the innate immune system defensive response to microorganisms, neutrophils generate huge amounts of reactive oxygen species (ROS), including superoxide anion (O_2_^−^), hydrogen peroxide (H_2_O_2_) and hypochlorous acid (HClO). Neutrophils also secrete cytokines such as IL-8 and enzymes degrading extracellular matrix (ECM) elastase and matrix metalloproteinases. These factors are mainly responsible for neutrophil infiltration and microbial destruction. On the other hand, the prolonged over-activation of neutrophils (due to exogenous bacterial factors such as LPS or due to endogenous IL-8 produced by local fibroblasts) results in host tissue damage and is believed to contribute to the development of destructive phases of diseases with an inflammatory background [5–8].

*Lythrum salicaria* belongs to the Lythraceae family. The main compounds on which pharmacopoeial standardization is based (according to the European Pharmacopoeia, 8th edition) are tannins. *C*-glucosidic monomeric (vescalagin and castalagin) and dimeric (salicarinins A, B, C) ellagitannins have been shown to be the dominant compounds in aqueous extracts [9, 10]. Other phenolics, such as *C*-glucosidic flavonoids, orientin, vitexin, isovitexin and iso-orientin, were also detected. In previous studies, the presence of phenolic acids, mainly ellagic acid together with phthalates and sterols, has been described [11]. Pawlaczyk et al. [12] isolated acidic glycoconjugate with pro-coagulant activity. However, since the *C*-glucosidic ellagitannins are significantly dominant, they were targeted in the present study as the compounds likely to be producing the aqueous extract’s bioactivity.

Because of excessive neutrophil infiltration and release of pro-inflammatory cytokines, enzymes degrading ECM and ROS are important in the development of inflammatory diseases of the skin and mucosa, and the ex-vivo model using neutrophils isolated from human peripheral venous blood was chosen to explain the beneficial effects of extracts of *Lythrum salicaria* and to evaluate the participation of ellagitannins in the observed activity.

## Materials and methods

### Plant material and chemicals

**Fig. 1:**
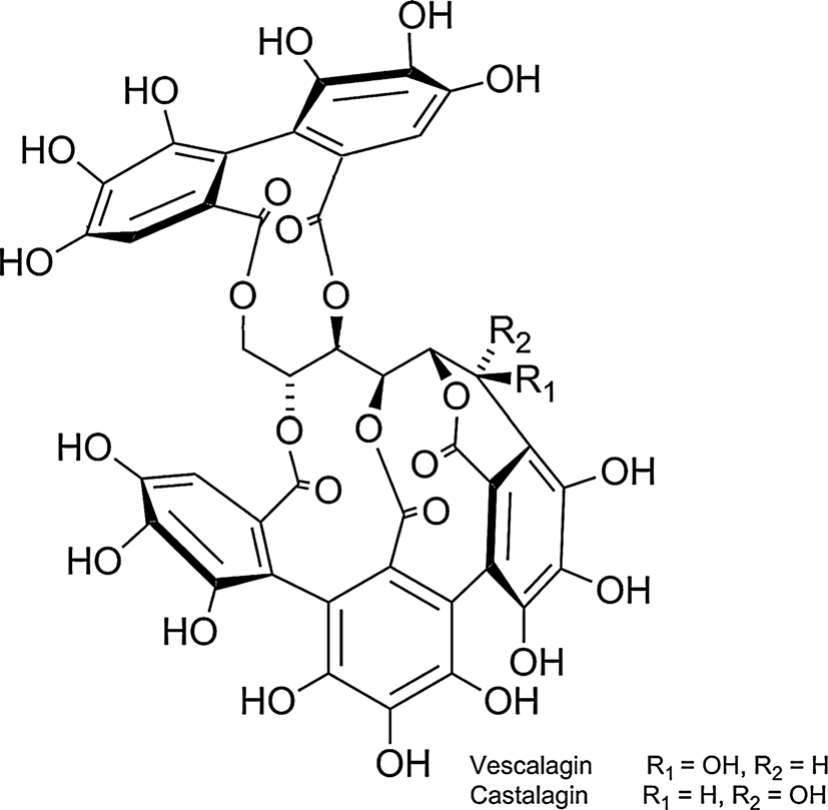
Structures of monomeric ellagitannins: vescalagin and castalagin

**Fig. 2:**
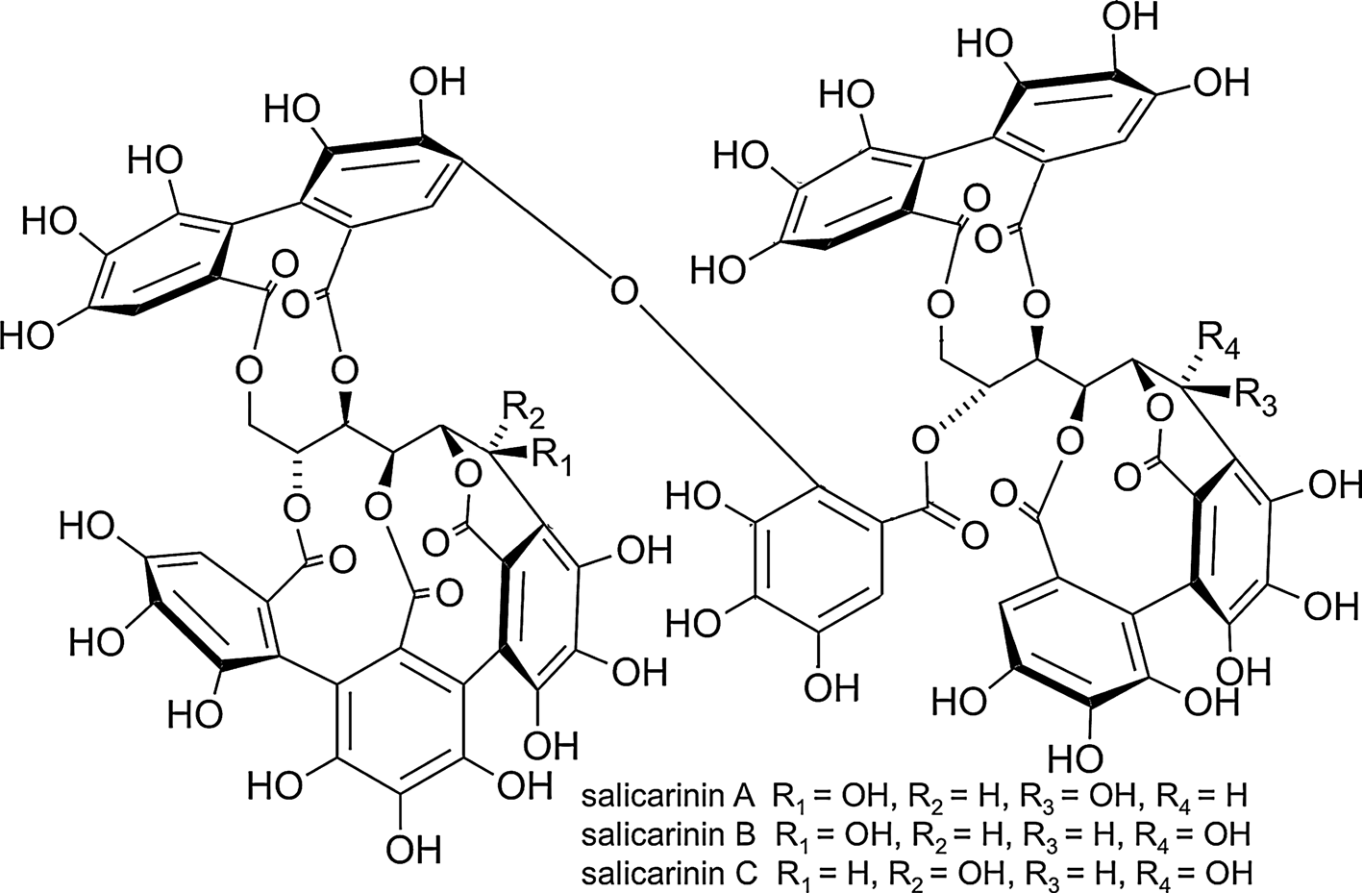
Structures of dimeric ellagitannins: salicarinin A, salicarinin B and salicarinin C

The *Lythrum salicaria* flowering herb was collected from its natural habitat in Umer in the Świętokrzyskie region (Poland) in July 2011. Its identity was confirmed anatomically and morphologically based on its pharmacopoeial monograph in the Department of Pharmacognosy and Molecular Basis of Phytotherapy, Medical University of Warsaw, where the voucher specimen UMER2011 is deposited. Preparation of an aqueous extract (LSH) and isolation of ellagitannins: vescalagin (V), castalagin (C), salicarinin A (SA), salicarinin B (SB) and salicarinin C (SC) (Figs. 1, 2) from *Lythrum salicaria* was performed according to Piwowarski and Kiss [9]. The identity of the ellagitannins was confirmed by NMR and MS spectra. Isolated ellagitannins were of >95 % purity (determined by the HPLC–DAD–CAD method). Camptothecin (98 % purity), luminol, PMA (4β-phorbol-12β-myristate-R13-acetate), f-MLP (formyl-met-leu-phenylalanine), ascorbic acid (reagent grade), curcumin, SAAVNA (*N*-succinyl-alanine-alanine-valinine-*p*-nitroanilide), LPS, cytochalasin A, TMB (3,3′,5, 5′-tetramethylbenzidine) liquid substrate system, Triton X-100, Hanks’ balanced salt solution (HBSS) and RPMI 1640 medium, bovine testis hyaluronidase and hyaluronic acid sodium salt from *Streptococcus equi* sp. were purchased from Sigma–Aldrich GmbH (Steinheim, Germany). Lucigenin and quercetin (>95 % purity) were purchased from Carl Roth (Karlsruhe, Germany). Gallic acid (>96 % purity) was purchased from ChromaDex (Santa Ana, USA). Propidium iodide was purchased from BD Biosciences (San Diego, CA, USA). All substances used were of >95 % purity. Phosphate-buffered saline (PBS) was purchased from Gibco (Carlsbad, CA, USA).

### Isolation of human neutrophils

Peripheral venous blood was taken from healthy human donors (20–35 years old) in the Warsaw Blood Donation Centre. Donors did not smoke and were not taking any medication. They were clinically confirmed to be healthy, and routine laboratory tests showed values within the normal ranges. The study conformed to the principles of the Declaration of Helsinki. Neutrophils were isolated using a standard method of dextran sedimentation prior to hypotonic lysis of erythrocytes and to centrifugation in a Ficoll Hypaque gradient. The purity of neutrophil preparations was >97 %, and viability measured by trypan blue exclusion was >98 %. Neutrophils were then resuspended in appropriate medium to perform the selected assay.

### Cytotoxicity

Cytotoxicity of the extract and ellagitannins was determined by standard flow cytometric probe using propidium iodide (PI) staining to distinguish cells with diminished membrane integrity according to the method previously described by Shinella et al. [13]. For short-term cytotoxicity determination, neutrophils (4 × 10^5^/mL) were incubated in PBS with extract at a concentration of 20 μg/mL and compounds at a concentration of 20 μM for 1.5 h. For long-term cytotoxicity, neutrophils (2 × 10^5^/mL) were cultured in a 24-well plate in RPMI 1640 medium with 10 % FBS, 10 mM HEPES, and 2 mM l-glutamine for 24 h at 37 °C with 5 % CO_2_ in the presence of extract at a concentration of 20 μg/mL and compounds at concentrations of 20 μM. After 24 h, the neutrophils were harvested and centrifuged (1500 RPM; 10 min; 4 °C), washed once with cold PBS and resuspended in 400 μL PBS. 5 μL of PI (50 μg/mL) solution was added to the cell suspension. After 15 min of incubation at room temperature, cells were analysed using a BD FACSCalibur flow cytometer (BD Biosciences, San Jose, CA, USA); 10,000 events were recorded per sample. Cells that displayed high permeability to PI were expressed as a percentage of PI(+) cells. Camptothecin at a concentration of 10 μM was used as a positive control.

### IL-8 and MMP-9 production

Neutrophils (2 × 10^5^/mL) were cultured in 24-well plates in RPMI 1640 medium with 10 % FBS, 10 mM HEPES, and 2 mM l-glutamine in the absence or presence of LPS (100 ng/mL) for 24 h at 37 °C with 5 % CO_2_ in the absence or presence of extract at concentrations of 1, 5 and 20 μg/mL, and compounds at concentrations of 1, 5 and 20 μM were added to 1 mL of cell suspension 1 h before the stimuli. After 24 h, the neutrophils were harvested and centrifuged (2000 RPM; 10 min; 4 °C). The amount of IL-8 or MMP-9 released into cell supernatants was measured by enzyme-linked immunosorbent assay (ELISA) following the manufacturer’s instructions (R&D Systems, Minneapolis, MN, USA). PBS was used as a non-stimulated control. Curcumin was used as a positive control, according to Aggarwal and Harikumar [14] and Antoine et al. [15].

### Expression of adhesion molecules CD62L and CD11b/CD18

The influence of urolithins at the level of adhesion molecules on the neutrophil surface was determined using the flow cytometric method. 500 μL of cell suspension (1 × 10^6^) in PBS buffer was incubated with extract at concentrations of 1, 5 and 20 μg/mL or compound at concentrations of 1, 5 and 20 μM for 30 min at 37 °C prior to 30 min stimulation with 10 μL of cytochalasin A (5 μg/mL) and f-MLP (0.1 μg/mL). Neutrophils were marked with monoclonal antibody against CD62L-(APC)-conjugate (Becton–Dickinson) or CD11b-(PE)-conjugate (Becton–Dickinson) and incubated for 30 min at 4 °C in the dark. The cells were analysed by flow cytometry FACSCalibur (Becton–Dickinson) and data from 20,000 events were recorded.

### Elastase release

Neutrophil elastase release was determined using SAAVNA as a substrate, and *p*-nitrophenol was measured spectrophotometrically. 100 μL of cell suspension (5 × 10^5^/mL) in HBSS was preincubated with 50 μL of compound or extract solution (final concentrations of 1, 5 and 20 μM or μg/mL) for 15 min at 37 °C and then stimulated with 50 μL of cytochalasin A (5 μg/mL) and f-MLP (0.1 μg/mL) for 15 min. The neutrophils were centrifuged (2000 rpm; 10 min; 4 °C). After the addition of 50 μL of SAAVNA solution (1.6 mg/mL) to 100 μL of supernatant, the extent of *p*-nitrophenol was measured spectrophotometrically for 1 h at intervals of 20 min, at 412 nm using a microplate reader (BioTek). According to Kanashiro et al. [16], quercetin was used as a positive control.

### Myeloperoxidase (MPO) release

Neutrophil MPO release was determined using TMB as a substrate. The assay is based on the oxidation of TMB by MPO in the presence of H_2_O_2_ [17]. 100 μL of cell suspension (2 × 10^5^/mL) in HBSS was preincubated with 50 μL of compound or extract solution (final concentrations of 1, 5 and 20 μM or μg/mL) for 15 min at 37 °C and then stimulated with 50 μL of cytochalasin A (5 μg/mL) and f-MLP (0.1 μg/mL) for 15 min. After centrifugation (2000 rpm; 10 min; 4 °C), 100 μL of supernatant was incubated with 50 μL of the TMB liquid substrate system. The reaction was terminated after 20 min by the addition of 2 M hydrochloric acid. The absorbance was measured at 655 nm using a microplate reader (BioTek). Gallic acid was used as a positive control, according to Kroes et al. [18].

### ROS production

Generation of oxidants by f-MLP or PMA-stimulated neutrophils was measured using luminol- or lucigenin-dependent chemiluminescence tests. 70 μL of cell suspension (2 × 10^5^/mL) in HBSS was incubated with 50 μL of compound or extract solution (final concentrations of 1, 5 and 20 μM or μg/mL) together with 50 μL of luminol (100 μM) or lucigenin (200 μM) solution. ROS production was initiated by the addition of 30 μL of f-MLP (0.1 μg/mL) or PMA (1 μg/mL). Changes in chemiluminescence at 37 °C were measured immediately for 45 min at intervals of 2 min in a microplate reader (Biotek). Ascorbic acid was used as a positive control.

### Hyaluronidase activity

The influence of extract and ellagitannins on hyaluronidase activity was measured using the USP turbidimetric method modified by Piwowarski et al. [19]. Heparin was used as a positive control.

### Statistical analysis

The results are presented as mean values ± SEM of the indicated number of experiments. Statistical significance of differences between means was determined by one-way ANOVA. For comparison of results with the control group, Dunnett’s post hoc test was used. To compare the differences between the inhibitory activities of compounds, Tukey’s post hoc test was performed. Results with a *p* value < 0.05 were considered statistically significant. All analyses were performed using Statistica 10 software.

## Results

### Cytotoxicity

*Lythrum salicaria* aqueous extract (LSH) as well as isolated ellagitannins at tested concentrations did not show any cytotoxic effect on neutrophils in either long- or short-term tests (Figs. S1, S2).

### IL-8 and MMP-9 production

**Fig. 3:**
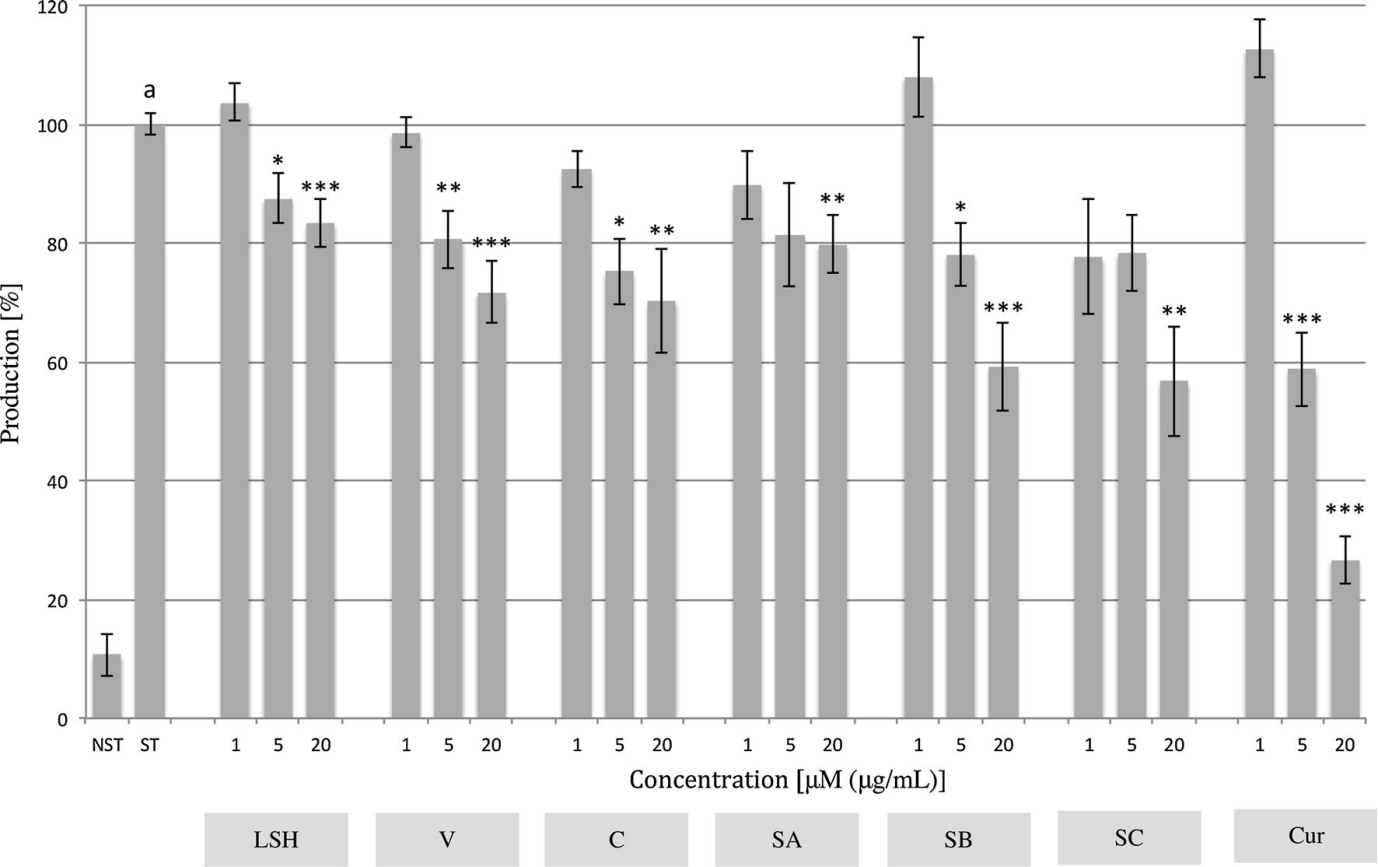
Effect of *Lythrum salicaria* aqueous extract (*LSH*) at concentrations of 1, 5 and 20 μg/mL and vescalagin (*V*), castalagin (*C*), salicarinin A, B and C (*SA*, *SB*, *SC*, respectively) at concentrations of 1, 5 and 20 μM on IL-8 production by LPS-stimulated neutrophils. Curcumin (*Cur*) at concentrations of 1, 5 and 20 μM was used as a positive control. Data were expressed as mean ± SEM of three separate experiments performed with neutrophils isolated from independent donors assayed in duplicate. Statistical significance: *p < 0.05, **p < 0.01, *** *p* < 0.001 versus stimulated control (Dunnett’s post hoc test); ^a^statistically significant (*p* < 0.001) versus non-stimulated control; *ST* stimulated control, *NST* non-stimulated control. Values of means, SEM and statistics are provided in Table S1

The influence of LSH and ellagitannins on the inflammatory response of neutrophils triggered by stimulation of Toll-like receptor-4 (TLR-4) by LPS was examined. LSH exhibited a moderate inhibitory effect towards IL-8 production (Fig. 3; Table S1). However, statistically significant inhibition of production was observed at concentrations of 5 and 20 μg/mL (12.5 ± 4.3 and 16.6 ± 4.2 %, respectively). Ellagitannins seem to be responsible for the observed inhibition, as all tested compounds have shown activity at a concentration of 20 μM. LSH at tested concentrations had no effect on production responsible for ECM degradation MMP-9 (Fig. S3; Table S2). However dimeric ellagitannins SB and SC were slightly active at a concentration of 20 μM (21.2 ± 4.1 and 13.4 ± 3.7 %, respectively). The effects of extract and ellagitannins on IL-8 and MMP-9 production were compared with curcumin used as a positive control.

### Expression of adhesion molecules, CD62L and CD11b/CD18

**Fig. 4:**
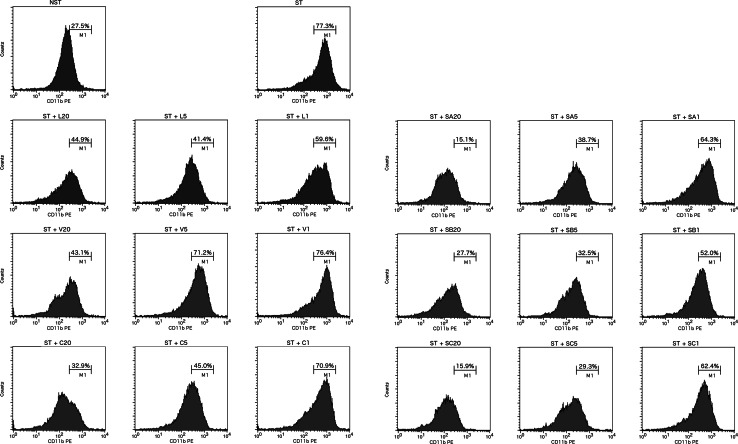
Effect of *Lythrum salicaria* aqueous extract (*LSH*) at concentrations of 1, 5 and 20 μg/mL and vescalagin (*V*), castalagin (*C*), salicarinin A, B and C (*SA*, *SB*, *SC*, respectively) at concentrations of 1, 5 and 20 μM on integrin CD11b expression on the surface of cytochalasin A/f-MLP-stimulated neutrophils. *ST* stimulated control, *NST* non-stimulated control, *M1* histogram marker for stimulated control

The rolling and the firm adhesion of the neutrophils to the endothelium leading to their transendothelial migration to the inflammation site are crucial events of inflammatory state progression. When the pro-inflammatory factor occurs, shedding of the molecule responsible for rolling, selectin (CD62L), is observed, while the level of the molecule providing firm adhesion, integrin (CD11b), becomes enhanced on the neutrophil surface [20]. LSH and ellagitannins have shown dose-dependent inhibition of CD11b expression triggered by cytochalasin A/f-MLP (Fig. 4). The effects of LSH were observed even at a concentration of 1 μg/mL. The greater contribution of dimeric ellagitannins to this activity was observed, as SA, SB and SC acted significantly more strongly than monomeric compounds. Neither LSH nor ellagitannins have prevented CD62L from shedding (data not shown).

### Elastase release

**Fig. 5:**
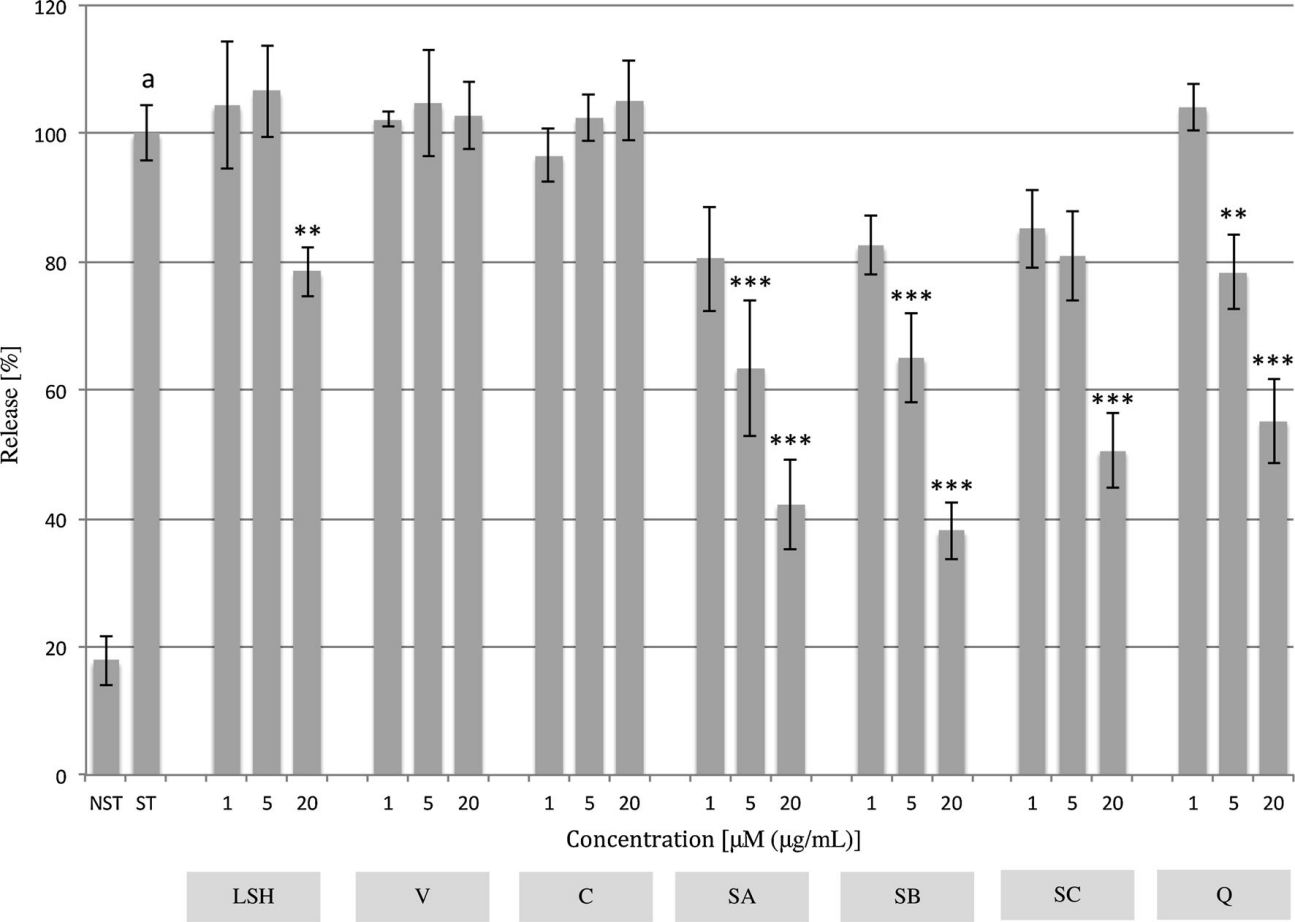
Effect of *Lythrum salicaria* aqueous extract (*LSH*) at concentrations of 1, 5 and 20 μg/mL and vescalagin (*V*), castalagin (*C*), salicarinin A, B and C (*SA*, *SB*, *SC*, respectively) at concentrations of 1, 5 and 20 μM on elastase release from cytochalasin A/f-MLP-stimulated neutrophils. Quercetin (*Q*) at concentrations of 1, 5 and 20 μM was used as a positive control. Data were expressed as mean ± SEM of four separate experiments performed with neutrophils isolated from independent donors assayed in duplicate. Statistical significance: *p < 0.05, **p < 0.01, *** *p* < 0.001 versus stimulated control (Dunnett’s post hoc test); ^a^statistically significant (*p* < 0.001) versus non-stimulated control; *ST* stimulated control, *NST* non-stimulated control. Values of means, SEM and statistics provided in Table S3

Elastase is a neutrophil serine proteinase responsible for ECM degradation, stored in azurophilic granules ready for immediate release upon stimulation. It increases tissue permeability and eases neutrophil migration to the inflammation site [21]. LSH was shown to inhibit elastase release from cytochalasin A/f-MLP stimulated neutrophils (Fig. 5; Table S3). Statistically significant inhibition was observed at 20 μg/mL (21.5 ± 3.9 %). Only dimeric ellagitannins, SA, SB and SC, were responsible for the observed effects, all potent to inhibit enzyme release at a concentration of 20 μM (57.8 ± 7.1, 61.8 ± 4.5 and 49.4 ± 5.9 % inhibition, respectively). The effect was compared with quercetin used as a positive control. Monomeric ellagitannins V and C were completely inactive at the tested concentrations.

### MPO release

**Fig. 6:**
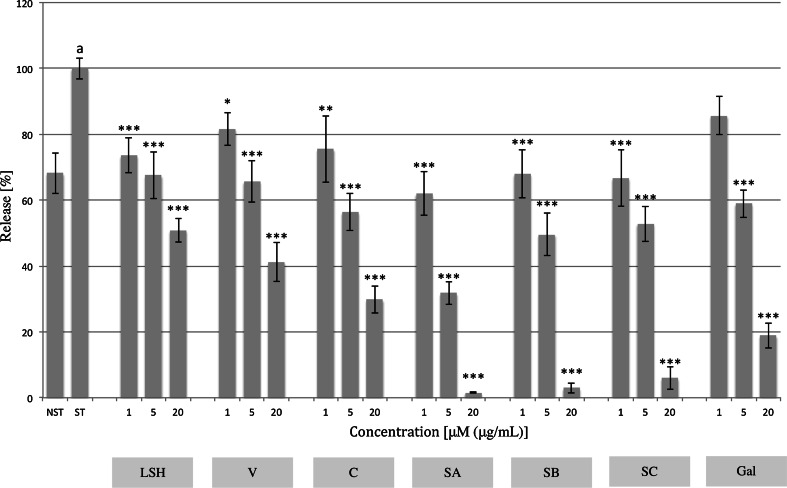
Effect of *Lythrum salicaria* aqueous extract (*LSH*) at concentrations of 1, 5 and 20 μg/mL and vescalagin (*V*), castalagin (*C*), salicarinin A, B and C (*SA*, *SB*, *SC*, respectively) at concentrations of 1, 5 and 20 μM on MPO release from cytochalasin A/f-MLP-stimulated neutrophils. Gallic acid (*Gal*) at concentrations of 1, 5 and 20 μM was used as a positive control. Data were expressed as mean ± SEM of three separate experiments performed with neutrophils isolated from independent donors assayed in triplicate. Statistical significance: *p < 0.05, **p < 0.01, *** *p* < 0.001 versus stimulated control (Dunnett’s post hoc test); ^a^statistically significant (*p* < 0.001) versus non-stimulated control; *ST* stimulated control, *NST* non-stimulated control. Values of means, SEM and statistics provided in Table S4

MPO is another pro-inflammatory ready-to-release enzyme stored in neutrophils’ azurophilic granules, being responsible for the production of HClO from H_2_O_2_ and Cl^−·^during respiratory bursts. LSH inhibited its release even at a concentration of 1 μg/mL (26.5 ± 5.4 % inhibition). Monomeric ellagitannins V and C expressed inhibitory activity at all tested concentrations. However, dimeric compounds SA, SB, and SC produced the highest contribution to this effect, showing inhibition at a concentration of 20 μM of 98.5 ± 0.3, 97.1 ± 1.5 and 94.0 ± 3.4 %, respectively. Their activity was compared with the known neutrophil MPO release inhibitor, gallic acid (Fig. 6; Table S4).

### ROS production

**Fig. 7:**
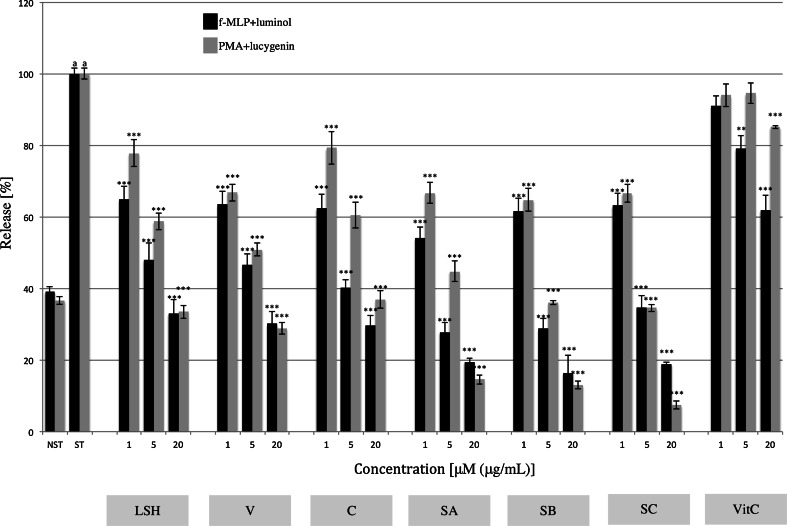
Effect of *Lythrum salicaria* aqueous extract (*LSH*) at concentrations of 1, 5 and 20 μg/mL and vescalagin (*V*), castalagin (*C*), salicarinin A, B and C (*SA*, *SB*, *SC*, respectively) at concentrations of 1, 5 and 20 μM on ROS release from neutrophils upon f-MLP or PMA stimulation detected by luminol or lucygenin, respectively. Ascorbic acid (*VitC*) at concentrations of 1, 5 and 20 μM was used as a positive control. Data were expressed as mean ± SEM of four separate experiments performed with neutrophils isolated from independent donors assayed in triplicate. Statistical significance: *p < 0.05, **p < 0.01, *** *p* < 0.001 versus stimulated control (Dunnett’s post hoc test); ^a^statistically significant (*p* < 0.001) versus non-stimulated control; *ST* stimulated control, *NST* non-stimulated control. Values of means, SEM and statistics provided in Table S5

The inhibition of ROS release from stimulated neutrophils was determined using two different models. One was with stimulation achieved by bacterial-derived peptide, f-MLP, which leads to generation of O_2_^–^, H_2_O_2_ and HclO, and the other was by direct kinase C activator, PMA, which results in O_2_^–^ and H_2_O_2_ release. LSH inhibited ROS release in a dose-dependent manner at all tested concentrations in both models, acting significantly more strongly than ascorbic acid (Fig. 7; Table S5). Monomeric and dimeric ellagitannins were responsible for the observed inhibition, and the latter were shown to be more potent in these assays.

### Hyaluronidase activity

Increased hyaluronidase activity is not only responsible for ECM degradation and increased tissue permeability, but also leads to the appearance of a low-molecular-weight hyaluronan which is known to be a strong inflammation inducer [22]. LSH was shown to inhibit hyaluronidase activity in a dose-dependent manner with IC_50_ = 10.1 ± 1.2 μg/mL (Fig. S4; Table S6), reaching 94.4 ± 0.6 % inhibition at 20 μg/mL. Ellagitannins were established to be responsible for this effect and, as in assays conducted on neutrophil models, dimeric SA, SB and SC (IC_50_ = 1.6 ± 0.1, 1.6 ± 0.2 and 2.5 ± 0.2 μM, respectively) acted more strongly than monomeric V and C (IC_50_ = 3.1 ± 0.2 and 3.1 ± 0.2 μM, respectively).

## Discussion

In our previous screening study conducted on tannin-rich plant materials used in Polish traditional medicine, *Lythrum salicaria* aqueous extract was shown to be the most potent for prevention of ECM degradation [19]. Strong inhibition of hyaluronidase activity and elastase release were determined together with primary phytochemical investigations. It was suspected that ellagitannins, which were present in the extract in significant amounts (27.4 ± 0.8 %), were responsible for these effects. To evaluate this hypothesis, initially the dominating ellagitannins were isolated and identified together with qualitative characterization of extract using the UHPLC–DAD–MS/MS method [9]. Three novel dimeric *C*-glucosidic ellagitannins, salicarinin A, B and C, were isolated in addition to two known ellagitannins, vescalagin and castalagin. The amount of each ellagitannin in *Lythrum salicaria* was determined using the UHPLC–CAD method to indicate their levels in aqueous extract. Vescalagin, castalagin, salicarinin A and salicarinin C were 10.22 ± 0.51, 7.12 ± 0.32, 11.2 ± 0.52 and 9.68 ± 0.49 mg/g of raw material, respectively [10].

The ex-vivo studies presented here were conducted using the neutrophil model and they revealed potent anti-inflammatory activity of *Lythrum salicaria* for which dominating *C*-glucosidic ellagitannins were shown to be mainly responsible. According to the results obtained, the dimeric salicarinins have a greater effect than the monomeric vescalagin and castalagin. The observed differences are consistent with previous studies concerned with the structure–activity relationships of ellagitannis, which demonstrated that dimeric compounds have significantly stronger biological effects than monomeric compounds [23]. In the case of elastase release, only dimeric salicarinins were responsible for the extract’s inhibitory effect. Hrenn et al. [24] conducted studies on pedunculagin (monomeric ellagitannin) and agrimoniin (dimeric ellagitannin) pointing to stronger elastase inhibition for the latter but in-silico studies led the authors to conclude that this effect occurs in an unspecific manner. Taking into consideration the lack of activity determined for monomeric vescalagin and castalagin compared with the strong activity of salicarinins, it can be concluded that the *C*-glucosidic ellagitannins examined here act specifically on neutrophil elastase. In the case of the anti-oxidant and anti-myeloperoxidase activity of *Lythrum salicaria* extract, the larger contribution is attributable also to dimeric salicarinins as, in these assays, their influence was determined to be stronger than that of monomeric compounds. However, inhibition by vescalagin and castalagin was also significantly marked. Similar observations were made for inhibition of CD11b expression as integrin is responsible for the firm adhesion of neutrophils to endothelium, which is the necessary first event for neutrophil transendothelial migration and infiltration to the inflammation site. Moreover, *Lythrum salicaria* extract inhibited hyaluronidase activity in a dose-dependent manner as did all isolated ellagitannins. As in neutrophil model assays, dimeric ellagitannins were more potent than monomeric ones. Inhibition of enzyme is known to be related to increased permeability of vascular and other tissues, together with further enhancement of the inflammatory process which can be beneficial from a therapeutic point of view. Taking into consideration the above results, it can be concluded that dimeric salicarinins A, B and C are mainly responsible for the anti-inflammatory activity of *Lythrum salicaria*, together with the weaker, but also active, monomeric vescalagin and castalagin. The orientation of the hydroxyl group at anomeric C1 of the glucose chain had no significant influence on activity either in dimeric or in monomeric compounds.

Studies conducted by Tunalier et al. [4] on an in-vivo animal model have shown anti-nociceptive (*p*-benzoquinone-induced abdominal constriction test) and anti-inflammatory (carrageenan-induced hind paw edema model) activity for orally administered polar extracts. The authors pointed out the flavonoids, isovitexin and isoorientin, as compounds responsible for the observed bioactivity. The contribution of ellagitannins needs further investigation due to their problematic bioavailability and their metabolism by gut microbiota to dibenzopyran-6-one derivatives, urolithins [25]. The anti-oxidant in-vitro effects observed on the neutrophil model are consistent with those determined by Tunalier et al. [4].

Castalagin was shown to have a gastroprotective effect in vivo. Administered orally at a dose of 50 mg/kg b.w., it was very potent in the reduction of ethanol-induced gastric lesions in a mouse model. The authors proposed that the antioxidant properties of tannins could be a mechanism of gastroprotective activity [26]. The influence on neutrophil pro-inflammatory activity determined in the present study could also make an important contribution as their infiltration and activation has been shown to play a part in the development of ethanol-induced gastric lesions [27].

The effects of *Lythrum salicaria* extract and isolated ellagitannins on neutrophil pro-inflammatory activity could explain and support its use in the treatment of diseases for which the development of neutrophil over-activation is responsible. Neutrophils have been shown to participate in the development and progression of various gastrointestinal diseases. Reactive oxygen species derived from neutrophils are implicated in the pathogenesis of oesophageal inflammation induced by the reflux of gastroduodenal contents [28]. Neutrophil elastase is involved in stress-induced gastric mucosal lesion formation by decreasing gastric PGI2 production, which leads to reduced gastric mucosal blood flow and subsequent ischaemic mucosal injury [29]. Elastase and ROS were also indicated as important contributors to the gastric mucosal injury induced by administration of aspirin during *Helicobacter pylori* infection [30]. It is thus indicated that local pharmacological inhibition of neutrophil elastase could help to prevent gastric mucosal injury of different aetiologies. Neutrophils are also crucial participants in inflammatory bowel disease. It has been shown that strong and specific IL-8 expression in the affected mucosa correlates with the histological grade of active inflammation. Secretion of IL-8 at sites of intestinal inflammation triggers the ongoing recruitment of neutrophils and leads to the release of proteases and ROS responsible for tissue damage and a vicious cycle of neutrophil attraction and activation. Neutrophil trafficking is currently highlighted in designing a therapy for inflammatory bowel disease targeting adhesion molecules, which are crucial for neutrophil intestinal recruitment and retention [31].

The contribution of neutrophils to the development of various skin and mucosa conditions is also underlined. Neutrophil-derived ROS are involved in the disruption of the integrity of the follicular epithelium, which is responsible for the inflammatory processes of acne [32]. Neutrophils play an important role in the pathogenesis of many autoimmune skin diseases by expressing cell-surface receptors and intracellular signalling molecules. Inhibition of their pro-inflammatory activity is a mechanism of many synthetic anti-inflammatory drugs and is a target for designing new molecules effective for therapy of skin autoimmune diseases [33]. Genetic and experimental data have shown a clear association between neutrophil infiltration into the periodontal tissues and the severity and progression of inflammatory periodontal diseases. Release of chemokines, elastase and ROS leads to vascular endothelium integrity damage, which increases vascular permeability, a cause of further tissue destruction and oedema [34].

The effectiveness of external use of *Lythrum salicaria* in traditional medicine in the treatment of skin and mucosa diseases can be additionally supported by its anti-microbial activity towards *Staphylococcus aureus*, *Proteus mirabilis* and *Micrococcus luteus*, which vescalagin has been shown to be responsible for [35]. Antifungal activity towards *Candida albicans* was also determined [36]. Moreover, the anticoagulant activity attributed to glycoconjugates present in *Lythrum salicaria* [12, 37] can further explain the benefits of its traditional use in such cases as haemorrhoidal disease or periodontosis and gingivitis.

The first complex cell-based anti-inflammatory studies of *Lythrum salicaria* have shown strong activity of the aqueous extract on stimulated neutrophils, the enhanced response of which is known to trigger pathological changes in skin and mucosa tissues. These observations can support and explain the traditional use of *Lythrum salicaria* for external therapy of certain diseases with an inflammatory background. *C*-glucosidic ellagitannins, especially dimeric salicarinins, were found to be the factors responsible for these effects.

## Electronic supplementary material

Below is the link to the electronic supplementary material.

Supplementary material 1 (DOCX 91 kb)

Supplementary material 2 (TIFF 213 kb)

Supplementary material 3 (TIFF 12559 kb)

Supplementary material 4 (TIFF 33970 kb)

Supplementary material 5 (TIFF 33970 kb)

Supplementary material 6 (DOCX 65 kb)

Supplementary material 7 (DOCX 58 kb)

Supplementary material 8 (DOCX 62 kb)

Supplementary material 9 (DOCX 68 kb)

Supplementary material 10 (DOCX 74 kb)

Supplementary material 11 (DOCX 72 kb)

## References

[1] Galgut P, Galgut N, Dowsett SA, Kowolik MJ (2001). Periodontics: current concepts and treatment strategies.

[2] Popovic Z, Smiljanic M, Matic R, Kostic M, Nikic P, Bojovic S (2012). Phytotherapeutical plants from the Deliblato sands (Serbia): traditional pharmacopoeia and implications for conservation. Indian J Tradit Knowl.

[3] Tita I, Mogosanu GD, Tita MG (2009). Ethnobotanical inventory of medicinal plants from the south-west of Romania. Farmacia.

[4] Tunalier Z, Kosar M, Kupeli E, Calis I, Baser KH (2007). Antioxidant, anti-inflammatory, anti-nociceptive activities and composition of *Lythrum salicaria* L. extracts. J Ethnopharm.

[5] Agaiby AD, Dyson M (1999). Immuno-inflammatory cell dynamics during cutaneous wound healing. J Anat.

[6] de Paula PR, Matos D, Franco M, Speranzini MB, Figueiredo F, de Santana ICB, Chacon-Silva MA, Bassi DG (2004). Why do anal wounds heal adequately? A study of the local immunoinflammatory defense mechanisms. Dis Colon Rectum.

[7] Dickson-Gonzalez SM, de Uribe ML, Rodriguez-Morales AJ (2009). Polymorphonuclear neutrophil infiltration intensity as consequence of *Entamoeba histolytica* density in amebic colitis. Surg Infect.

[8] Stvrtinova V, Jahnova E, Weissova S, Horvathova M, Ferencik M (2001). Inflammatory mechanisms involving neutrophils in chronic venous insufficiency of lower limbs. Bratisl Lek Listy.

[9] Piwowarski JP, Kiss AK (2013). *C*-glucosidic ellagitannins from Lythri herba (European Pharmacopoeia): chromatographic profile and structure determination. Phytochem Anal.

[10] Granica S, Piwowarski JP, Kiss AK (2014). Determination of *C*-glucosidic ellagitannins in Lythri herba by ultra-high performance liquid chromatography coupled with charged aerosol detector: method development and validation. Phytochem Anal.

[11] Rauha JP, Wolfender JL, Salminen JP, Pihlaja K, Hostettmann K, Vuorela H (2001). Characterization of the polyphenolic composition of purple loosestrife (*Lythrum salicaria*). Z Naturforsch C.

[12] Pawlaczyk I, Czerchawski L, Kanska J, Bijak J, Capek P, Pliszczak-Krol A, Gancarz R (2010). An acidic glycoconjugate from *Lythrum salicaria* L. with controversial effects on haemostasis. J Ethnopharm.

[13] Schinella G, Aquila S, Dade M (2008). Anti-inflammatory and apoptotic activities of pomolic acid isolated from *Cecropia pachystachya*. Planta Med.

[14] Aggarwal BB, Harikumar KB (2009). Potential therapeutic effects of curcumin, the anti-inflammatory agent, against neurodegenerative, cardiovascular, pulmonary, metabolic, autoimmune and neoplastic diseases. Int J Biochem Cell Biol.

[15] Antoine F, Simard JC, Girard D (2013). Curcumin inhibits agent-induced human neutrophil functions in vitro and lipopolysaccharide-induced neutrophilic infiltration in vivo. Int Immunopharm.

[16] Kanashiro A, Souza JG, Kabeya LM, Azzolini AE, Lucisano-Valim YM (2007). Elastase release by stimulated neutrophils inhibited by flavonoids: importance of the catechol group. Z Naturforsch C.

[17] Suzuki K, Ota H, Sasagawa S, Sakatani T, Fujikura T (1983). Assay method for myeloperoxidase in human polymorphonuclear leukocytes. Anal Biochem.

[18] Kroes BH, van den Berg AJ, Quarles van Ufford HC, van Dijk H, Labadie RP (1992). Anti-inflammatory activity of gallic acid. Planta Med.

[19] Piwowarski JP, Kiss AK, Kozlowska-Wojciechowska M (2011). Anti-hyaluronidase and anti-elastase activity screening of tannin-rich plant materials used in traditional Polish medicine for external treatment of diseases with inflammatory background. J Ethnopharm.

[20] Liu JJ, Song CW, Yue Y, Duan CG, Yang J, He T, He YZ (2005). Quercetin inhibits LPS-induced delay in spontaneous apoptosis and activation of neutrophils. Inflammation Res.

[21] Siedle B, Hrenn A, Merfort I (2007). Natural compounds as inhibitors of human neutrophil elastase. Planta Med.

[22] Stern R, Asari AA, Sugahara KN (2006). Hyaluronan fragments: an information-rich system. Eur J Cell Biol.

[23] Coca A, Feldman KS, Lawlor MD, Quideau S (2009). Immunomodulatory ellagitannin chemistry. Chemistry and biology of ellagitannins.

[24] Hrenn A, Steinbrecher T, Labahn A, Schwager J, Schempp CM, Merfort I (2006). Plant phenolics inhibit neutrophil elastase. Planta Med.

[25] Gonzalez-Barrio R, Truchado P, Ito H, Espin JC, Tomas-Barberan FA (2011). UV and MS identification of urolithins and nasutins, the bioavailable metabolites of ellagitannins and ellagic acid in different mammals. J Agric Food Chem.

[26] Khennouf S, Benabdallah H, Gharzouli K, Amira S, Ito H, Kim TH, Yoshida T, Gharzouli A (2003). Effect of tannins from *Quercus suber* and *Quercus coccifera* leaves on ethanol-induced gastric lesions in mice. J Agric Food Chem.

[27] Tanaka K, Tanaka Y, Suzuki T, Mizushima T (2011). Protective effect of beta-(1,3→1,6)-d-glucan against irritant-induced gastric lesions. Br J Nutr.

[28] Yamaguchi T, Yoshida N, Tomatsuri N, Takayama R, Katada K, Takagi T, Ichikawa H, Naito Y, Okanoue T, Yoshikawa T (2005). Cytokine-induced neutrophil accumulation in the pathogenesis of acute reflux esophagitis in rats. Int J Mol Med.

[29] Harada N, Okajima K, Liu W, Uchiba M (2000). Activated neutrophils impair gastric cytoprotection role of neutrophil elastase. Dig Dis Sci.

[30] Yoshida N, Sugimoto N, Ochiai J, Nakamura Y, Ichikawa H, Naito Y, Yoshikawa T (2002). Role of elastase and active oxygen species in gastric mucosal injury induced by aspirin administration in *Helicobacter pylori*-infected Mongolian gerbils. Aliment Pharmacol Ther.

[31] Brazil JC, Louis NA, Parkos CA (2013). The role of polymorphonuclear leukocyte trafficking in the perpetuation of inflammation during inflammatory bowel disease. Inflamm Bowel Dis.

[32] Akamatsu H, Horio T (1998). The possible role of reactive oxygen species generated by neutrophils in mediating acne inflammation. Dermatology.

[33] Nemeth T, Mocsai A (2012). The role of neutrophils in autoimmune diseases. Immunol Lett.

[34] Scott DA, Krauss J (2012). Neutrophils in periodontal inflammation. Front Oral Biol.

[35] Becker H, Scher JM, Speakman JB, Zapp J (2005). Bioactivity guided isolation of antimicrobial compounds from *Lythrum salicaria*. Fitoterapia.

[36] Rauha JP, Remes S, Heinonen M, Hopia A, Kahkonen M, Kujala T, Pihlaja K, Vuorela H, Vuorela P (2000). Antimicrobial effects of Finnish plant extracts containing flavonoids and other phenolic compounds. Int J Food Microbiol.

[37] Pawlaczyk I, Capek P, Czerchawski L, Bijak J, Lewik-Tsirigotis M, Pliszczak-Krol A, Gancarz R (2011). An anticoagulant effect and chemical characterization of *Lythrum salicaria* L. glycoconjugates. Carbohydr Polym.

