# Supplementary material for: Contribution of C-glucosidic ellagitannins to Lythrum salicaria L. influence on pro-inflammatory functions of human neutrophils
Source: J Nat Med. 2014 Oct 28;69(1):100–10. doi: 10.1007/s11418-014-0873-5 (PMC4544630; doi:10.1007/s11418-014-0873-5)
Supplement: Supplementary file 1 — Supplementary material 1 (DOCX 91 kb) [file 11418_2014_873_MOESM1_ESM.docx]

**Fig. S1.**

Histograms presenting 24 h cytotoxicity tests of *Lythrum salicaria* L. herb aqueous extract (LSH) at concentration of 20 μg/mL, vescalagin, castalagin, salicarinin A, B and C at concentration of 20 μM using propidium iodide (PI) staining to distinguish cells with diminished membrane integrity. Camptothecin at concentration of 10 μM was used as a positive control.

**Fig. S2.**

Histograms presenting 1.5 h cytotoxicity tests of *Lythrum salicaria* L. herb aqueous extract (LSH) at concentration of 20 μg/mL, vescalagin, castalagin, salicarinin A, B and C at concentration of 20 μM using propidium iodide (PI) staining to distinguish cells with diminished membrane integrity.

**Fig. S3.**

Effect of *Lythrum salicaria* L. aqueous extract (LSH) at concentration of 1, 5 and 20 μg/mL and vescalagin (V), castalagin (C), salicarinin A, B and C (SA, SB, SC respectively) at concentration of 1, 5 and 20 μM on MMP-9 production by LPS stimulated neutrophils. Curcumin (Cur) at concentration of 1, 5 and 20 μM was used as a positive control. Data were expressed as mean ± SEM of three separate experiments performed with neutrophils isolated from independent donors assayed in duplicate. Statistical significance: **p*<0.05, ***p*<0.01, *** *p*<0.001 versus stimulated control (Dunnett’s *post hoc* test); a- statistically significant (*p*<0.001) versus non-stimulated control; ST- stimulated control; NST- non-stimulated control. Values of means, SEM and statistics provided in Table S2.

**Fig. S4.**

Effect of *Lythrum salicaria* L. aqueous extract (LSH) at concentration of 1, 2, 5 and 20 μg/mL and vescalagin (V), castalagin (C), salicarinin A, B and C (SA, SB, SC respectively) at concentration of 1, 2, 5 and 20 μM on hyaluronidase activity. Heparin (Hep) at concentration of 1, 2, 5 and 20 μg/mL was used as a positive control. Data were presented as mean ± SEM of three independent experiments assayed in triplicate. Statistical significance: **p*<0.05, ***p*<0.01, *** *p*<0.001 versus control (Dunnett’s *post hoc* test). Values of means, SEM and statistics provided in Table S6.

**Table S1.**

Effect of *Lythrum salicaria* L. aqueous extract (LSH) at concentration of 1, 5 and 20 μg/mL and vescalagin (V), castalagin (C), salicarinin A, B and C (SA, SB, SC respectively) at concentration of 1, 5 and 20 μM on IL-8 production by LPS stimulated neutrophils. Curcumin (Cur) at concentration of 1, 5 and 20 μM was used as a positive control. Data were expressed as mean ± SEM of three separate experiments performed with neutrophils isolated from independent donors assayed in duplicate. ST- stimulated control; NST- non-stimulated control. Statistical significance was determined using Dunnett’s *post hoc* test with stimulated cells established as a control.

**Table S2.**

Effect of *Lythrum salicaria* L. aqueous extract (LSH) at concentration of 1, 5 and 20 μg/mL and vescalagin (V), castalagin (C), salicarinin A, B and C (SA, SB, SC respectively) at concentration of 1, 5 and 20 μM on MMP-9 production by LPS stimulated neutrophils. Curcumin (Cur) at concentration of 1, 5 and 20 μM was used as a positive control. Data were expressed as mean ± SEM of three separate experiments performed with neutrophils isolated from independent donors assayed in duplicate. ST- stimulated control; NST- non-stimulated control. Statistical significance was determined using Dunnett’s *post hoc* test with stimulated cells established as a control.

**Table S3.**

Effect of *Lythrum salicaria* L. aqueous extract (LSH) at concentration of 1, 5 and 20 μg/mL and vescalagin (V), castalagin (C), salicarinin A, B and C (SA, SB, SC respectively) at concentration of 1, 5 and 20 μM on elastase release from cytohalasin A/f-MLP stimulated neutrophils. Quercetin (Q) at concentration of 1, 5 and 20 μM was used as a positive control. Data were expressed as mean ± SEM of four separate experiments performed with neutrophils isolated from independent donors assayed in duplicate. ST- stimulated control; NST- non-stimulated control. Statistical significance was determined using Dunnett’s *post hoc* test with stimulated cells established as a control.

**Table S4.**

Effect of *Lythrum salicaria* L. aqueous extract (LSH) at concentration of 1, 5 and 20 μg/mL and vescalagin (V), castalagin (C), salicarinin A, B and C (SA, SB, SC respectively) at concentration of 1, 5 and 20 μM on MPO release from cytohalasin A/f-MLP stimulated neutrophils. Gallic acid (Gal) at concentration of 1, 5 and 20 μM was used as a positive control. Data were expressed as mean ± SEM of three separate experiments performed with neutrophils isolated from independent donors assayed in triplicate. ST- stimulated control; NST- non-stimulated control. Statistical significance was determined using Dunnett’s *post hoc* test with stimulated cells established as a control.

**Table S5.**

Effect of *Lythrum salicaria* L. aqueous extract (LSH) at concentration of 1, 5 and 20 μg/mL and vescalagin (V), castalagin (C), salicarinin A, B and C (SA, SB, SC respectively) at concentration of 1, 5 and 20 μM on ROS release from neutrophils upon f-MLP or PMA stimulation detected by luminol or lucygenin respectively. Ascorbic acid (VitC) at concentration of 1, 5 and 20 μM was used as a positive control. Data were expressed as mean ± SEM of four separate experiments performed with neutrophils isolated from independent donors assayed in triplicate. ST- stimulated control; NST- non-stimulated control. Statistical significance was determined using Dunnett’s *post hoc* test with stimulated cells established as a control.

**Table S6.**

Effect of *Lythrum salicaria* L. aqueous extract (LSH) at concentration of 1, 2, 5 and 20 μg/mL and vescalagin (V), castalagin (C), salicarinin A, B and C (SA, SB, SC respectively) at concentration of 1, 2, 5 and 20 μM on hyaluronidase activity. Heparin (Hep) at concentration of 1, 2, 5 and 20 μg/mL was used as a positive control. Data were presented as mean ± SEM of three independent experiments assayed in triplicate. Statistical significance was determined using Dunnett’s *post hoc* with 100% active enzyme established as a control.
