# Supplementary figures and images for: Contribution of C-glucosidic ellagitannins to Lythrum salicaria L. influence on pro-inflammatory functions of human neutrophils
Source: J Nat Med. 2014 Oct 28;69(1):100–10. doi: 10.1007/s11418-014-0873-5 (PMC4544630; doi:10.1007/s11418-014-0873-5)

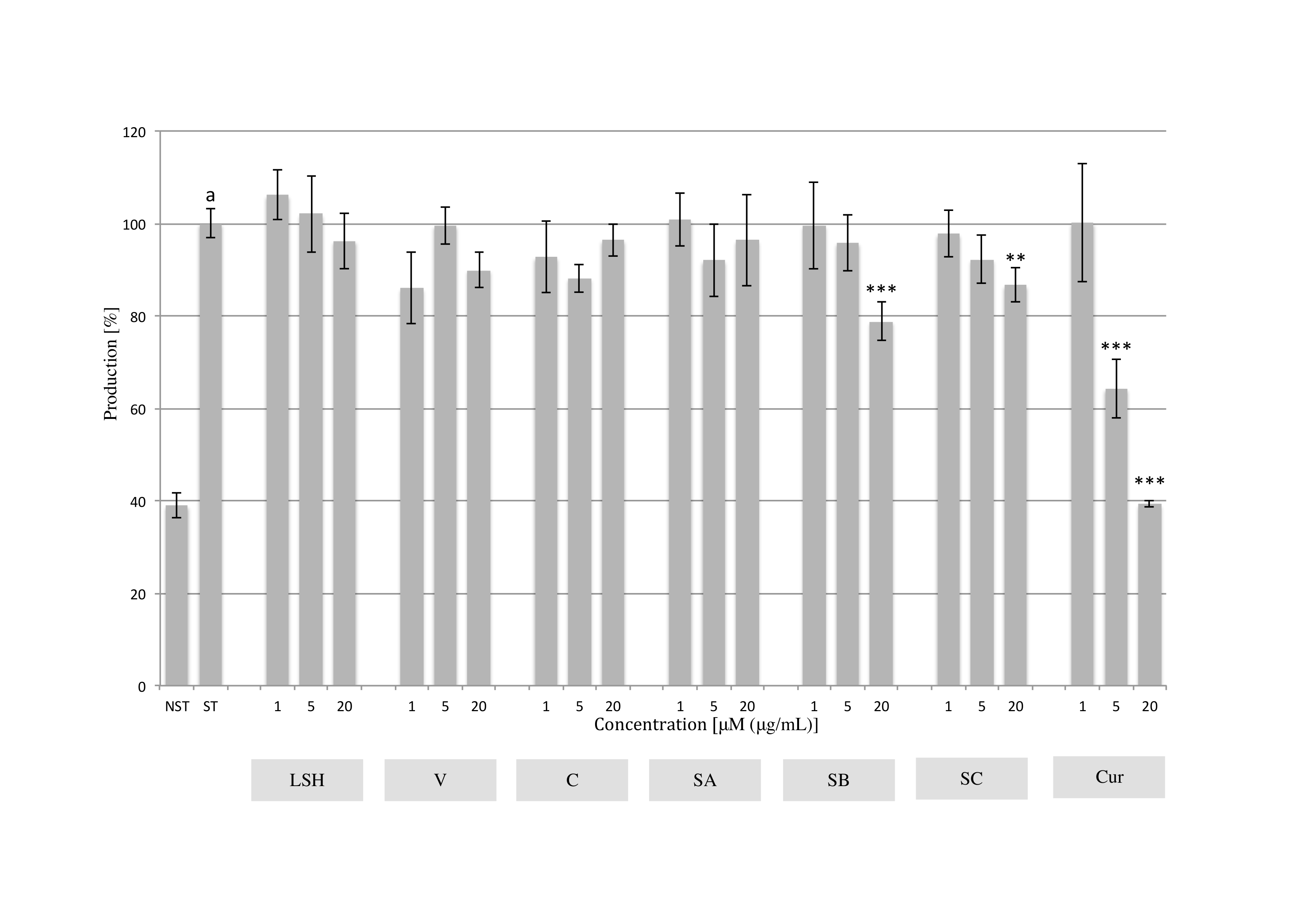

Supplement: Supplementary file 4 — Supplementary material 4 (TIFF 33970 kb) [file 11418_2014_873_MOESM4_ESM.tiff]

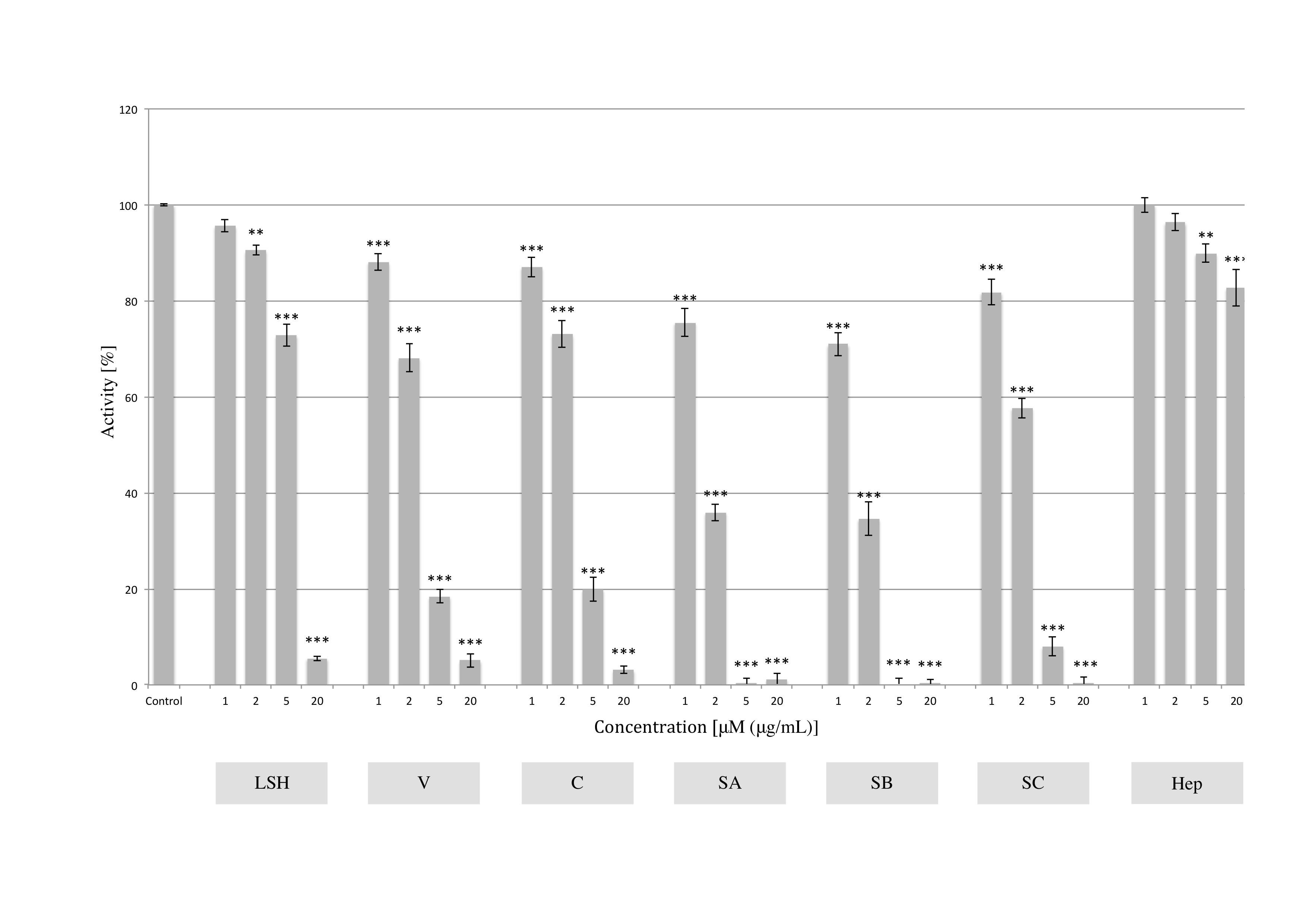

Supplement: Supplementary file 5 — Supplementary material 5 (TIFF 33970 kb) [file 11418_2014_873_MOESM5_ESM.tiff]
