# Supplementary material for: Contribution of C-glucosidic ellagitannins to Lythrum salicaria L. influence on pro-inflammatory functions of human neutrophils
Source: J Nat Med. 2014 Oct 28;69(1):100–10. doi: 10.1007/s11418-014-0873-5 (PMC4544630; doi:10.1007/s11418-014-0873-5)
Supplement: Supplementary file 6 — Supplementary material 6 (DOCX 65 kb) [file 11418_2014_873_MOESM6_ESM.docx]

| IL-8 production |  |  |  |
| --- | --- | --- | --- |
|  | Mean (%) | ±SEM | *p* value (Dunnett's test) |
| NST | **10,71** | 3,57 | 0,000020 |
| ST | **100,00** | 1,76 | control |
|  |  |  |  |
| L1 | **103,69** | 3,07 | 0,994296 |
| L5 | **87,50** | 4,21 | 0,017314 |
| L20 | **83,38** | 4,18 | 0,000884 |
|  |  |  |  |
| V1 | **98,64** | 2,51 | 0,637424 |
| V5 | **80,63** | 4,86 | 0,009446 |
| V20 | **71,68** | 5,17 | 0,000100 |
|  |  |  |  |
| C1 | **92,43** | 3,04 | 0,376363 |
| C5 | **75,24** | 5,55 | 0,010102 |
| C20 | **70,24** | 8,81 | 0,004583 |
|  |  |  |  |
| SA1 | **89,82** | 5,77 | 0,259927 |
| SA5 | **81,38** | 8,66 | 0,068827 |
| SA20 | **79,80** | 4,95 | 0,003662 |
|  |  |  |  |
| SB1 | **107,98** | 6,72 | 0,946748 |
| SB5 | **78,12** | 5,33 | 0,017392 |
| SB20 | **59,17** | 7,40 | 0,000125 |
|  |  |  |  |
| SC1 | **77,70** | 9,64 | 0,829150 |
| SC5 | **78,29** | 6,43 | 0,097906 |
| SC20 | **56,68** | 9,17 | 0,001981 |
|  |  |  |  |
| Cur1 | **112,66** | 4,85 | 0,993034 |
| Cur5 | **58,75** | 6,19 | 0,000036 |
| Cur20 | **26,70** | 4,02 | 0,000024 |
