# Supplementary material for: Contribution of C-glucosidic ellagitannins to Lythrum salicaria L. influence on pro-inflammatory functions of human neutrophils
Source: J Nat Med. 2014 Oct 28;69(1):100–10. doi: 10.1007/s11418-014-0873-5 (PMC4544630; doi:10.1007/s11418-014-0873-5)
Supplement: Supplementary file 7 — Supplementary material 7 (DOCX 58 kb) [file 11418_2014_873_MOESM7_ESM.docx]

| MMP-9 production |  |  |  |
| --- | --- | --- | --- |
|  | Mean (%) | ±SEM | *p* value (Dunnett's test) |
| NST | **38,97** | 2,72 | 0,000020 |
| ST | **100,00** | 3,07 | control |
|  |  |  |  |
| L1 | **106,11** | 5,41 | 0,999877 |
| L5 | **102,03** | 8,38 | 0,997109 |
| L20 | **96,27** | 6,06 | 0,950874 |
|  |  |  |  |
| V1 | **85,96** | 7,75 | 0,456437 |
| V5 | **99,57** | 4,03 | 0,988994 |
| V20 | **89,91** | 3,76 | 0,679522 |
|  |  |  |  |
| C1 | **92,70** | 7,72 | 0,820289 |
| C5 | **88,18** | 3,05 | 0,559947 |
| C20 | **96,42** | 3,48 | 0,950646 |
|  |  |  |  |
| SA1 | **100,80** | 5,67 | 0,994306 |
| SA5 | **92,00** | 7,79 | 0,782652 |
| SA20 | **96,35** | 9,82 | 0,945925 |
|  |  |  |  |
| SB1 | **99,56** | 9,37 | 0,988718 |
| SB5 | **95,83** | 6,07 | 0,941749 |
| SB20 | **78,81** | 4,10 | 0,000274 |
|  |  |  |  |
| SC1 | **97,74** | 5,02 | 0,974854 |
| SC5 | **92,17** | 5,15 | 0,811369 |
| SC20 | **86,61** | 3,68 | 0,008285 |
|  |  |  |  |
| Cur1 | **100,14** | 12,79 | 0,991674 |
| Cur5 | **64,26** | 6,32 | 0,000437 |
| Cur20 | **39,26** | 0,73 | 0,000020 |
