# Supplementary material for: Contribution of C-glucosidic ellagitannins to Lythrum salicaria L. influence on pro-inflammatory functions of human neutrophils
Source: J Nat Med. 2014 Oct 28;69(1):100–10. doi: 10.1007/s11418-014-0873-5 (PMC4544630; doi:10.1007/s11418-014-0873-5)
Supplement: Supplementary file 8 — Supplementary material 8 (DOCX 62 kb) [file 11418_2014_873_MOESM8_ESM.docx]

| Elastase release | |  |  |
| --- | --- | --- | --- |
|  | Mean(%) | ±SEM | *p* value (Dunnett's test) |
| NST | **17,85** | 3,91 | 0,000020 |
| ST | **100,00** | 4,32 | control |
|  |  |  |  |
| L1 | **104,41** | 9,86 | 0,109650 |
| L5 | **106,59** | 7,12 | 0,998589 |
| L20 | **78,48** | 3,86 | 0,002079 |
|  |  |  |  |
| V1 | **102,25** | 1,24 | 0,989858 |
| V5 | **104,61** | 8,28 | 0,996812 |
| V20 | **102,89** | 5,29 | 0,992588 |
|  |  |  |  |
| C1 | **96,55** | 4,11 | 0,923406 |
| C5 | **102,40** | 3,53 | 0,991761 |
| C20 | **105,22** | 6,26 | 0,997860 |
|  |  |  |  |
| SA1 | **80,47** | 8,14 | 0,173613 |
| SA5 | **63,45** | 10,62 | 0,000678 |
| SA20 | **42,22** | 7,04 | 0,000020 |
|  |  |  |  |
| SB1 | **82,54** | 4,51 | 0,200716 |
| SB5 | **65,03** | 6,94 | 0,000172 |
| SB20 | **38,16** | 4,49 | 0,000020 |
|  |  |  |  |
| SC1 | **85,10** | 6,02 | 0,394716 |
| SC5 | **80,91** | 6,94 | 0,119373 |
| SC20 | **50,60** | 5,83 | 0,000020 |
|  |  |  |  |
| Q1 | **104,09** | 3,59 | 0,920218 |
| Q5 | **78,40** | 5,86 | 0,006744 |
| Q20 | **55,22** | 6,61 | 0,000023 |
