# Supplementary material for: Contribution of C-glucosidic ellagitannins to Lythrum salicaria L. influence on pro-inflammatory functions of human neutrophils
Source: J Nat Med. 2014 Oct 28;69(1):100–10. doi: 10.1007/s11418-014-0873-5 (PMC4544630; doi:10.1007/s11418-014-0873-5)
Supplement: Supplementary file 9 — Supplementary material 9 (DOCX 68 kb) [file 11418_2014_873_MOESM9_ESM.docx]

| MPO release |  |  |  |
| --- | --- | --- | --- |
|  | Mean(%) | ±SEM | *p* value (Dunnett's test) |
| NST | **68,20** | 6,19 | 0,000563 |
| ST | **100,00** | 3,19 | control |
|  |  |  |  |
| L1 | **73,55** | 5,35 | 0,000755 |
| L5 | **67,53** | 7,04 | 0,000060 |
| L20 | **50,86** | 3,58 | 0,000020 |
|  |  |  |  |
| V1 | **81,67** | 5,02 | 0,045323 |
| V5 | **65,70** | 6,28 | 0,000032 |
| V20 | **41,17** | 5,85 | 0,000020 |
|  |  |  |  |
| C1 | **75,53** | 10,08 | 0,007740 |
| C5 | **56,44** | 5,65 | 0,000020 |
| C20 | **29,85** | 4,10 | 0,000020 |
|  |  |  |  |
| SA1 | **61,96** | 6,70 | 0,000020 |
| SA5 | **31,79** | 3,43 | 0,000020 |
| SA20 | **1,52** | 0,22 | 0,000020 |
|  |  |  |  |
| SB1 | **67,99** | 7,39 | 0,000173 |
| SB5 | **49,60** | 6,41 | 0,000020 |
| SB20 | **2,93** | 1,47 | 0,000020 |
|  |  |  |  |
| SC1 | **66,67** | 8,52 | 0,000430 |
| SC5 | **52,85** | 5,38 | 0,000020 |
| SC20 | **5,95** | 3,38 | 0,000020 |
|  |  |  |  |
| gal1 | **85,62** | 5,79 | 0,418154 |
| gal5 | **58,97** | 4,21 | 0,000043 |
| gal20 | **18,88** | 3,77 | 0,000020 |
