# Supplementary material for: Contribution of C-glucosidic ellagitannins to Lythrum salicaria L. influence on pro-inflammatory functions of human neutrophils
Source: J Nat Med. 2014 Oct 28;69(1):100–10. doi: 10.1007/s11418-014-0873-5 (PMC4544630; doi:10.1007/s11418-014-0873-5)
Supplement: Supplementary file 10 — Supplementary material 10 (DOCX 74 kb) [file 11418_2014_873_MOESM10_ESM.docx]

| ROS release |  |  |  |  |  |  |  |  |
| --- | --- | --- | --- | --- | --- | --- | --- | --- |
|  |  |  |  |  |  |  |  |  |
| PMA + lucygenin |  |  |  |  | f-MLP + luminol |  |  |  |
|  | Mean(%) | ±SEM | *p* value (Dunnett's test) |  |  | Mean(%) | ±SEM | *p* value (Dunnett's test) |
| NST | **36,69** | 1,10 | 0,000021 |  | NST | **39,14** | 1,42 | 0,000021 |
| ST | **100,00** | 1,49 | control |  | ST | **100,00** | 1,53 | control |
|  |  |  |  |  |  |  |  |  |
| L1 | **77,86** | 3,81 | 0,000021 |  | L1 | **64,93** | 3,58 | 0,000021 |
| L5 | **58,79** | 2,34 | 0,000021 |  | L5 | **48,07** | 4,53 | 0,000021 |
| L20 | **33,52** | 1,85 | 0,000021 |  | L20 | **33,04** | 3,90 | 0,000021 |
|  |  |  |  |  |  |  |  |  |
| V1 | **66,84** | 2,38 | 0,000021 |  | V1 | **63,47** | 3,75 | 0,000021 |
| V5 | **50,90** | 1,77 | 0,000021 |  | V5 | **46,61** | 2,94 | 0,000021 |
| V20 | **28,93** | 1,68 | 0,000021 |  | V20 | **30,13** | 3,34 | 0,000021 |
|  |  |  |  |  |  |  |  |  |
| C1 | **79,28** | 4,52 | 0,000021 |  | C1 | **62,41** | 3,89 | 0,000021 |
| C5 | **60,48** | 3,55 | 0,000021 |  | C5 | **40,10** | 2,35 | 0,000021 |
| C20 | **37,00** | 2,48 | 0,000021 |  | C20 | **29,55** | 3,04 | 0,000021 |
|  |  |  |  |  |  |  |  |  |
| SA1 | **66,68** | 2,90 | 0,000021 |  | SA1 | **54,14** | 3,10 | 0,000021 |
| SA5 | **44,80** | 2,83 | 0,000021 |  | SA5 | **27,84** | 2,70 | 0,000021 |
| SA20 | **14,59** | 1,32 | 0,000021 |  | SA20 | **19,49** | 0,93 | 0,000021 |
|  |  |  |  |  |  |  |  |  |
| SB1 | **64,77** | 3,14 | 0,000021 |  | SB1 | **61,64** | 3,45 | 0,000021 |
| SB5 | **36,09** | 0,57 | 0,000021 |  | SB5 | **28,73** | 2,93 | 0,000021 |
| SB20 | **13,06** | 1,09 | 0,000021 |  | SB20 | **16,39** | 4,88 | 0,000021 |
|  |  |  |  |  |  |  |  |  |
| SC1 | **66,62** | 2,53 | 0,000021 |  | SC1 | **63,16** | 3,36 | 0,000021 |
| SC5 | **34,56** | 1,07 | 0,000021 |  | SC5 | **34,71** | 3,23 | 0,000021 |
| SC20 | **7,37** | 1,12 | 0,000021 |  | SC20 | **18,92** | 0,58 | 0,000021 |
|  |  |  |  |  |  |  |  |  |
| VitC1 | **93,99** | 3,14 | 0,075588 |  | VitC1 | **91,10** | 2,60 | 0,145049 |
| VitC5 | **94,53** | 2,81 | 0,106169 |  | VitC5 | **79,14** | 3,72 | 0,002077 |
| VitC20 | **85,15** | 0,43 | 0,000278 |  | VitC20 | **61,91** | 4,11 | 0,000028 |
