# Supplementary material for: Contribution of C-glucosidic ellagitannins to Lythrum salicaria L. influence on pro-inflammatory functions of human neutrophils
Source: J Nat Med. 2014 Oct 28;69(1):100–10. doi: 10.1007/s11418-014-0873-5 (PMC4544630; doi:10.1007/s11418-014-0873-5)
Supplement: Supplementary file 11 — Supplementary material 11 (DOCX 72 kb) [file 11418_2014_873_MOESM11_ESM.docx]

| Hyaluronidase activity |  |  |  |
| --- | --- | --- | --- |
|  | Mean(%) | ±SEM | *p* value (Dunnett's test) |
|  |  |  |  |
| Control | **100,00** | 0,20 |  |
|  |  |  |  |
| L1 | **95,65** | 1,29 | 0,453817 |
| L2 | **90,49** | 1,01 | 0,008355 |
| L5 | **72,84** | 2,28 | 0,000021 |
| L20 | **5,56** | 0,50 | 0,000021 |
|  |  |  |  |
| V1 | **88,10** | 1,71 | 0,000123 |
| V2 | **68,10** | 2,93 | 0,000021 |
| V5 | **18,39** | 1,40 | 0,000021 |
| V20 | **5,10** | 1,40 | 0,000021 |
|  |  |  |  |
| C1 | **87,07** | 2,09 | 0,000045 |
| C2 | **73,08** | 2,74 | 0,000021 |
| C5 | **19,92** | 2,48 | 0,000021 |
| C20 | **3,13** | 0,80 | 0,000021 |
|  |  |  |  |
| SA1 | **75,50** | 2,88 | 0,000021 |
| SA2 | **35,96** | 1,75 | 0,000021 |
| SA5 | **0,50** | 0,83 | 0,000021 |
| SA20 | **1,13** | 1,19 | 0,000021 |
|  |  |  |  |
| SB1 | **70,96** | 2,38 | 0,000021 |
| SB2 | **34,62** | 3,46 | 0,000021 |
| SB5 | **-0,07** | 1,55 | 0,000021 |
| SB20 | **0,50** | 0,75 | 0,000021 |
|  |  |  |  |
| SC1 | **81,82** | 2,69 | 0,000021 |
| SC2 | **57,68** | 2,11 | 0,000021 |
| SC5 | **8,00** | 1,93 | 0,000021 |
| SC20 | **0,50** | 1,05 | 0,000021 |
|  |  |  |  |
| Hep1 | **100,00** | 1,62 | 0,999832 |
| Hep2 | **96,35** | 1,81 | 0,764843 |
| Hep5 | **89,89** | 1,93 | 0,001422 |
| Hep20 | **82,73** | 3,81 | 0,000234 |
